# Direct comparison of tadalafil with sildenafil for the treatment of erectile dysfunction: a systematic review and meta-analysis

**DOI:** 10.1007/s11255-017-1644-5

**Published:** 2017-07-24

**Authors:** Binbin Gong, Ming Ma, Wenjie Xie, Xiaorong Yang, Yongming Huang, Ting Sun, Yanping Luo, Jiao Huang

**Affiliations:** Department of Urology, The First Affiliated Hospital of Nanchang University, Nanchang, 330000 Jiangxi Province China

**Keywords:** Tadalafil, Sildenafil, Erectile dysfunction, Meta-analysis, Systematic review

## Abstract

**Aims:**

Erectile dysfunction (ED) is a major care problem worldwide. Tadalafil and sildenafil are the two most common phosphodiesterase 5 inhibitors used to treat ED. This systematic review and meta-analysis were conducted to directly compare tadalafil with sildenafil for the treatment of ED.

**Methods:**

We designed a strategy for searching the PubMed, Embase, EBSCO, Web of Science and Cochrane library databases; the reference lists of the retrieved studies were also investigated. A literature review was performed to identify all published randomized or non-randomized controlled trials that compared tadalafil with sildenafil for the treatment of ED and to assess the quality of the studies. Two investigators independently and blindly screened the studies for inclusion. The meta-analysis was performed using RevMan 5.0.

**Results:**

A total of 16 trials that compared tadalafil with sildenafil for the treatment of ED were included in the meta-analysis. In the meta-analysis, tadalafil and sildenafil appeared to have similar efficacies and overall adverse event rates. However, compared with sildenafil, tadalafil significantly improved psychological outcomes. Furthermore, the patients and their partners preferred tadalafil over sildenafil, and no significant difference was found in the adherence and persistence rates between tadalafil and sildenafil. Additionally, the myalgia and back pain rates were higher and the flushing rate was lower with tadalafil than with sildenafil.

**Conclusion:**

Tadalafil shares a similar efficacy and safety with sildenafil and significantly improves patients’ sexual confidence. Furthermore, patients and their partners prefer tadalafil to sildenafil. Hence, tadalafil may be a better choice for ED treatment.

## Introduction

Erectile dysfunction (ED) is defined as the inability to achieve and maintain an erection sufficient to permit satisfactory sexual intercourse. ED is one of the most common sexual dysfunctions, and an estimated 5–20% of men are affected by moderate to severe ED around the world [1]. The prevalence of ED is approximately 15.77% in southern India, 15.0–49.5% in China, 56.1% in Iran and 58.9% in south-western Nigeria [2–5]. The estimated global prevalence has been increasing, and approximately 322 million men worldwide could be affected by ED by the year 2025 [6]. Although ED is a benign disorder, it can affect physical and psychosocial health and may have a significant impact on the quality of life of patients and their partners.

Currently, several treatment strategies are available for patients with ED, including non-invasive and invasive options. Oral phosphodiesterase type 5 inhibitors (PDE5-Is) are the first-line therapy for ED [7]. PDE5-Is are similar to cyclic guanosine monophosphate (cGMP) in structure; thus, PED5-Is can bind to PDE5 competitively and inhibit cGMP hydrolysis, leading to a penile erection [8]. Four PDE5-Is (sildenafil, tadalafil, vardenafil and avanafil) are currently approved by the US FDA and have comparable efficacy and side effect profiles [9]. Sildenafil and tadalafil are the two most common phosphodiesterase inhibitors (PDEIs) around the world. Sildenafil, the original PDEI, was introduced in 1998. Sildenafil has a quick onset of action of 30 min after the initial dose, a duration of action of 4–6 h and a maximum duration of 12 h [10]. Sildenafil is effective for the treatment of ED, although 20–50% of patients who respond to sildenafil discontinue its use [11]. Tadalafil is a selective, long-acting PDEI that was released in 2003. Tadalafil has an onset of 20 min and should be taken 30 min prior to intercourse; additionally, this drug has the longest duration of action in its class and a maximum duration of 72 h. A total of 52% of patients can have successful intercourse within 30 min of taking tadalafil [12]. Recently, several systematic reviews and meta-analyses have comparatively analysed the role of PDEIs, including sildenafil and tadalafil, in the treatment of ED. However, because most of these review articles were indirect comparative analyses, the differences between sildenafil and tadalafil remain unclear [13–15]. Hence, this meta-analysis was conducted to directly compare sildenafil with tadalafil for the treatment of ED and to provide guidance for the clinical treatment of ED.

## Materials and methods

### Search strategy

**Fig. 1:**
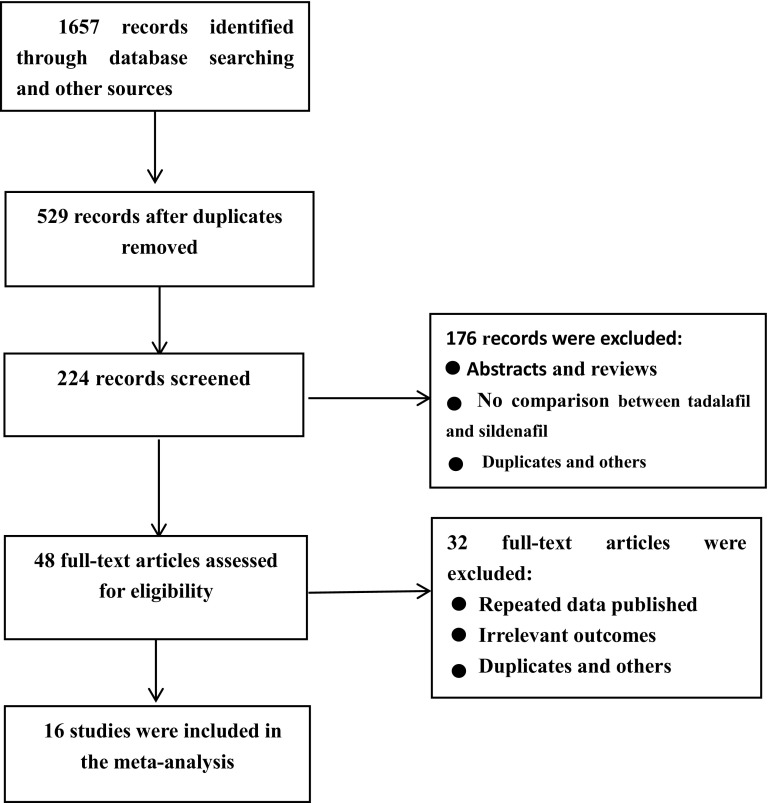
Flow chart of study selection

In January 2017, the PubMed, EBSCO, Web of Science, Cochrane library and Embase databases were searched for randomized or non-randomized controlled clinic trials of sildenafil and tadalafil. The search was performed by combining the term “erectile dysfunction or sexual dysfunction” with the following words describing the drug: tadalafil or Cialis and sildenafil or Viagra. We also searched for additional relevant studies by examining the reference lists of the selected papers and reviews. The search process was not limited by language, country or year of publication. The search strategy is presented in Fig. 1.

### Study selection

For the present systematic review, randomized or non-randomized controlled trials that met the following criteria were included: (a) the study included a comparison between sildenafil and tadalafil; (b) the study provided accurate data that applied to a meta-analysis, including the International Index of Erectile Function (IIEF)-EF domain, preference and treatment–emergent adverse events (TEAEs); and (c) the full text of the study could be acquired. When the same study was published in different journals or in different years, the most recent version was used for the meta-analysis. Abstracts, reviews and articles that did not contain accurate relevant data and a comparative analysis of sildenafil and tadalafil were excluded.

### Quality assessment

Two independent reviewers evaluated all identified publications, and any disagreement between reviewers was resolved by a third reviewer. The methodological quality was assessed using the Jadad score, with a Jadad score ≥3 indicating a high-quality article [16].

### Data extraction

Data extractions were performed by one reviewer and checked by a second reviewer. Information was extracted from the studies that met the above-mentioned inclusion criteria using a structured form. The first author, publication year, sample size, therapy that the patients received and assessment indices, including the IIEF, the men’s preference, the Self-Esteem and Relationship questionnaire (SEAR), total Erectile Dysfunction Inventory of Treatment Satisfaction (EDITS) scores and TEAEs, were collected for each study.

### Statistical analysis

A meta-analysis of comparable data was performed using RevMan 5.0. Statistical heterogeneity between studies was assessed using the *I* ^2^ statistic. Pronounced heterogeneity was indicated by a *P* value ≤0.05 and an *I* ^2^ value ≥50%; in these cases, a randomized effects model was executed. A *P* value ≥0.05 and *I* ^2^ ≤ 50 indicated no obvious heterogeneity between the studies, and a fixed effects model was chosen for the meta-analysis. Continuous data are presented as weighted mean differences (WMDs), and odds ratios (ORs) were calculated for dichotomy [both with 95% confidence intervals (CIs)]. Egger’s test was performed to evaluate the risk of publication bias. Additionally, a sensitivity analysis was performed to assess the stability of the outcome when low-quality and highly heterogeneous trials were included in the analysis.

## Results

### Search results

A total of 1657 articles were identified through the electronic databases and the manual review of the reference lists of the included studies. Based on the search strategy, a total of 16 studies were included in the review [17–31]. A total of 5189 patients were recruited in the final meta-analysis, and the mean patient age ranged from 36.9 to 56.2 years. Seven articles were open-label, randomized, multicentre, controlled studies; nine of the studies had a crossover design; and the other studies were prospective and observational. All the studies compared sildenafil and tadalafil. The common instruments used to assess these two PDEIs (sildenafil and tadalafil) were the IIEF-EF, preference, TEAEs, adherence and persistence. The characteristics of the studies are listed in Table 1.

**Table 1:** Characteristics of the included studies

Author	Year	Number	Intervention	Control	Assessment	Jadad score
Bai et al.	2016	383	Tadalafil: 20 mg	Sildenafil: 100 mg	preference, TEAEs	2
Buvat et al.	2013	770	Tadalafil: 5 mg OaD, 10 mg PRN	Sildenafil: 50 mg PRN	IIEF score, TEAEs	2
Helen et al.	2008	100	Tadalafil: 20 mg	Sildenafil: 100 mg	Preference, IIEF-EF, TEAEs	2
Eardley et al.	2005	291	Tadalafil: 10 mg, 20 mg	Sildenafil: 25,50,100 mg	Preference, IIEF, TEAEs	2
Govier et al.	2003	215	Tadalafil: 20 mg	Sildenafil: 50 mg	Preference, TEAEs	3
Hatzimouratids et al.	2014	770	Tadalafil: 2.5, 5 mg OaD; 10, 20 mg PRN	Sildenafil: 25, 50, 100 mg PRN	IIEF	3
Rubio-Aurioles et al.	2012	418	Tadalafil: 5 mg OaD, 20 mg PRN	Sildenafil: 100 mg PRN	IIEF, TEAEs	2
Rodriguez Tolra et al.	2006	90	Tadalafil: 20 mg	Sildenafil: 50 mg, vardenafil: 20 mg	IIEF, preference, TEAEs	2
Von Keitz et al.	2004	265	Tadalafil: 20 mg	Sildenafil: 50 mg	preference, TEAEs	3
Li et al.	2016	63	tadalafil: 20 mg	sildenafil: 100 mg	preference, IIEF	2
Cairol et al.	2014	104	Tadalafil	Sildenafil, vardenafil, lodenafil	IIEF, persistence and adherence	2
Stroberg et al.	2003	147	Tadalafil: 20 mg	Sildenafil: 25, 50, 100 mg	preference, TEAEs	2
Ahn et al.	2007	160	Tadalafil: 20 mg	Sildenafil: 50, 100 mg	preference, TEAEs	2
El-Meliegy et al.	2013	493	Tadalafil	Sildenafil, vardenafil	persistence, adherence, IIEF,	2
Taylor et al.	2009	409	Tadalafil	Sildenafil, vardenafil	TEAEs	2
Rubio-Aurioles et al.	2013	511	Tadalafil	Sildenafil, vardenafil	persistence, adherence IIEF	2

*TEAE* treatment–emergent adverse events, *IIEF* International Index of Erectile Function, *OaD* once a day, *PRN* on demand

### Efficacy measures

**Fig. 2:**
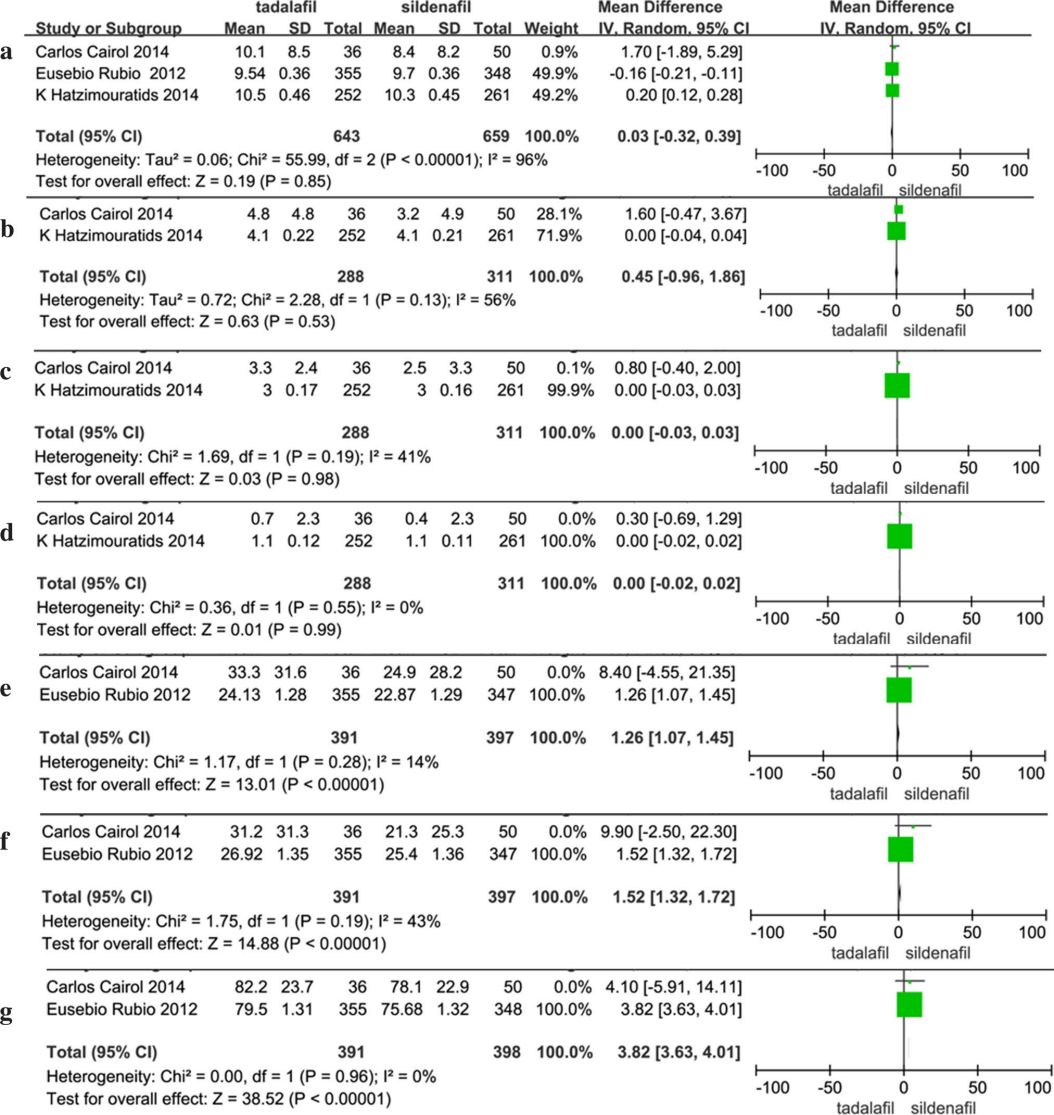
Forest plot of continuous variables: **a** IIEF-EF; **b** IIEF intercourse satisfaction; **c** IIEF overall satisfaction; **d** IIEF sexual desire; **e** SEAR confidence; **f** SEAR sexual relationship; **g** EDITS total

A meta-analysis was conducted to examine the efficacy of sildenafil and tadalafil. No significant differences were found in the changes in the IIEF-EF between patients who used sildenafil and those who used tadalafil (random effects model: WMD: 0.03, 95% CI: −0.32 to 0.39, *P* = 0.85). The pooled mean effect size of the IIEF intercourse satisfaction result for tadalafil versus sildenafil was 0.45 (95% CI: −0.96 to 1.86, *P* = 0.53). Our analysis found heterogeneity among the trials, and a random effects model was adopted. Similarly, for IIEF overall satisfaction and IIEF sexual desire, the pooled mean effect sizes were 0.00, 95% CI: −0.03 to 0.03, *P* = 0.98 and 0.00, 95% CI: −0.02 to 0.02, *P* = 0.99, respectively; neither of these studies exhibited evidence of heterogeneity. Compared with sildenafil, tadalafil significantlly improved the SEAR Confidence (fixed effects model: WMD: 1.26, 95% CI: 1.04–1.45, *P* < 0.00001), SEAR Sexual Relationship (fixed effects model: WMD: 1.52, 95% CI: 1.32–1.72, *P* < 0.00001) and EDITS total scores (fixed effects model: WMD: 3.82, 95% CI: 3.63–4.01, *P* < 0.00001; Table 2; Fig. 2).

**Table 2:** Meta-analysis of continuous variables

Index	Study	Heterogeneity test		Test for overall effect		Egger’s test
		*I* ^2^ (%)	*P*	WMD (95% CI)	*P*	*P*
IIEF-EF	3	96	<0.00001	0.03 [−0.32 to 0.39]	0.85	0.783
IIEF intercourse satisfaction	2	56	0.13	0.45 [−0.96 to 1.86]	0.53	–
IIEF overall satisfaction	2	41	0.19	0.00 [−0.03 to 0.03]	0.98	–
IIEF sexual desire	2	0	0.55	0.00 [−0.02 to 0.02]	0.99	–
SEAR confidence	2	14	0.28	1.26 [1.04–1.45]	<0.00001	–
SEAR sexual relationship	2	43	0.19	1.52 [1.32–1.72]	<0.00001	–
EDITS total	2	0	0.93	3.82 [3.63–4.01]	<0.00001	–

*IIEF* International Index of Erectile Function, *SEAR* Self-Esteem and Relationship questionnaire, *EDITS* Erectile Dysfunction Inventory of Treatment Satisfaction

### Preference, adherence and persistence

**Fig. 3:**
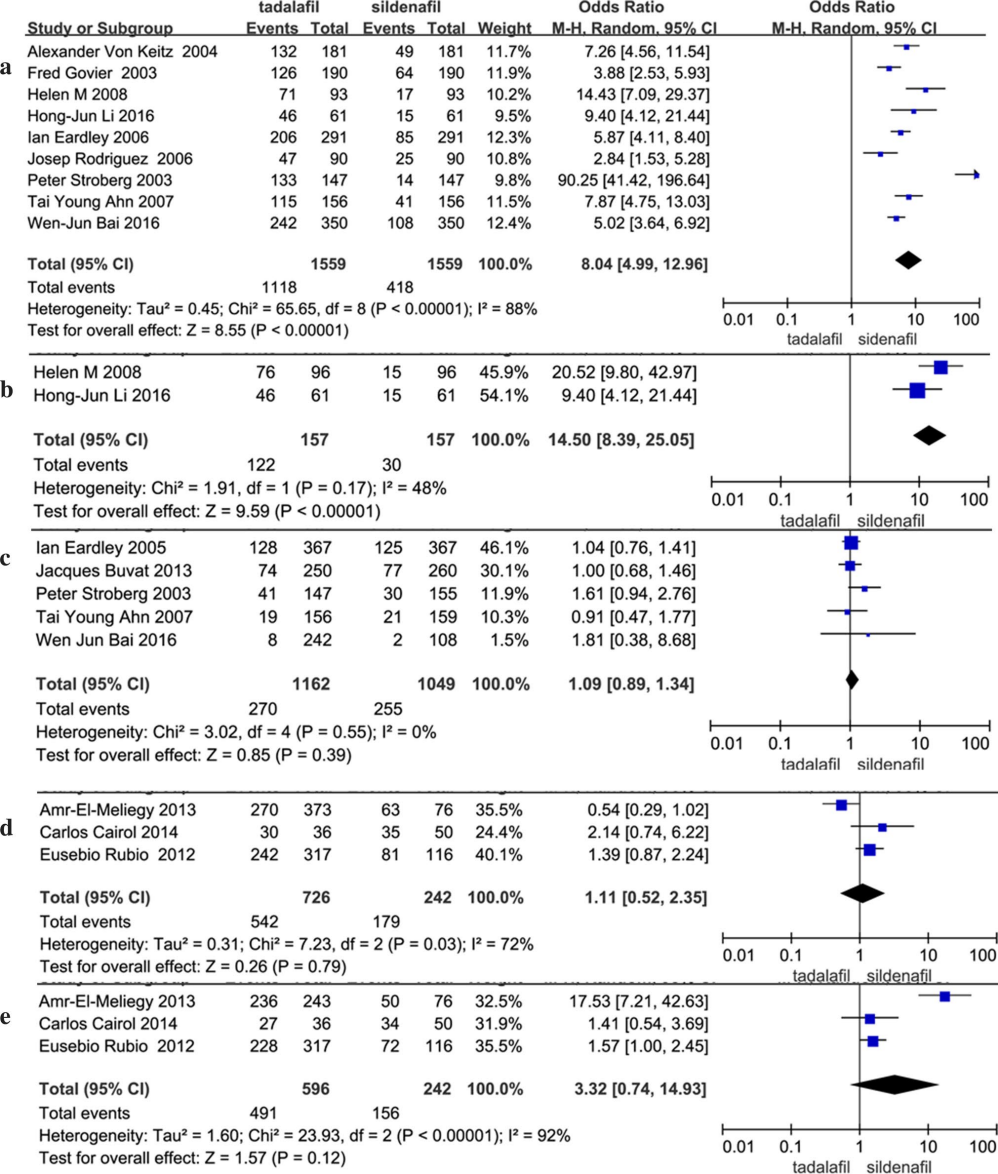
Forest plot of dichotomy: **a** preference—man; **b** preference—women; **c** adverse event; **d** adherence 3 months; **e** adherence 6 months

Nine of the studies included data regarding the men’s preference of tadalafil or sildenafil. According to our analysis, substantial heterogeneity was present in the data (*I* ^2^ = 88%, *P* < 0.00001). The pooled estimate of the OR was 8.04, and the 95% CI was 4.99–12.96 (*P* < 0.00001). This result suggests that men preferred tadalafil over sildenafil. Women also tended to choose tadalafil over sildenafil to cure erectile dysfunction (fixed effects model: OR: 14.50, 95% CI: 8.39–25.05, *P* < 0.00001). Three articles included 3-month adherence, 6-month adherence, 3-month persistence and 6-month persistence data, and were analysed via meta-analysis. According to our analysis, no evidence of heterogeneity was found among the trials, and there was no significant difference between tadalafil and sildenafil in terms of adherence and persistence (Table 3; Fig. 3).

**Table 3:** Meta-analysis of dichotomy

Index	Study	Heterogeneity test		Test for overall effect		Egger’s test
		*I* ^2^ (%)	*P*	OR (95% CI)	*P*	*P*
Preference—man	9	88	<0.00001	8.04 [4.99–12.96]	<0.00001	0.129
Preference—women	2	48	0.17	14.50 [8.39–25.05]	<0.00001	–
Adverse event	5	0	0.55	1.09 [0.89–1.34]	0.39	0.059
Adverse event—headache	12	37	0.09	0.97 [0.79–1.19]	0.77	0.002
Adverse event—myalgia	4	0	0.51	4.89 [1.66–14.43]	0.004	–
Adverse event—back pain	5	0	0.44	1.79 [1.06–3.02]	0.03	0.229
Adverse event—dyspepsia	7	0	0.76	1.41 [0.98–2.03]	0.06	0.106
Adverse event—flushing	10	0	0.87	0.39 [0.27–0.54]	<0.00001	0.002
Adverse event—nasal congestion	7	0	0.86	0.69 [0.45–1.06]	0.09	0.014
Adverse event—nasopharyngitis	3	5	0.35	0.93 [0.52–1.67]	0.8	0.042
Adherence 3 months	3	72	0.03	1.11 [0.52–2.35]	0.79	0.889
Adherence 6 months	3	92	<0.00001	3.32 [0.74–14.93]	0.12	0.622
Persistence 3 months	3	66	0.05	1.29 [0.66–2.53]	0.46	0.77
Persistence 6 months	3	27	0.25	1.33 [0.97–1.83]	0.08	0.843

### TEAEs

The most common TEAEs in patients treated with oral PDEIs included overall adverse events, headache, myalgia, back pain, dyspepsia, flushing, nasal congestion and nasopharyngitis. All the data were dichotomized, and ORs were calculated. For all studies, the random effects OR for the adverse events ratio of tadalafil versus sildenafil was 1.09 (95% CI: 0.89–1.34, *P* = 0.39), suggesting that the tadalafil and sildenafil groups shared a similar overall adverse events ratio. A total of 12 studies included headache data, and the pooled estimate of the OR was 0.97 (95% CI: 0.79–1.19, *P* = 0.77). The OR of myalgia for tadalafil compared with sildenafil based on the fixed effects model meta-analysis was larger (OR 4.89, 95% CI: 1.66–14.43, *P* = 0.004). Five articles contained back pain data, with no evidence of heterogeneity (*I* ^2^ = 0%, *P* = 0.44), and the pooled mean size for tadalafil versus sildenafil was 1.79 (95% CI: 1.06–3.02, *P* = 0.03). When comparing patients who suffered from flushing, significant differences were found between the tadalafil and sildenafil groups (fixed effects model: OR 0.39, 95% CI: 0.27–0.54, *P* < 0.00001). Other adverse events included dyspepsia, nasal congestion and nasopharyngitis, but no significant difference was found for these parameters between the tadalafil and sildenafil groups. The sensitivity analysis that excluded the above studies indicated that the pooled effect remained significantly different, and the direction of the forest plot did not change. Thus, the analysis results were stable (Table 3; Fig. 3).

### Publication bias

Egger’s test was conducted to assess the presence of possible publication bias. No evidence of publication bias was found for the IIEF-EF, preference, overall adverse events, back pain, dyspepsia, nasopharyngitis, adherence and persistence (Egger’s test *P* ≥ 0.05). The Egger’s test results found publication bias for headache, flushing and nasal congestion (*P* ≤ 0.05). The other parameters were not evaluated using Egger’s test because only two studies included relevant data (Tables 2, 3; Figs. 2, 3). Study quality was evaluated with the Jadad scoring system, and the results are shown in Table 1.

## Discussion

According to a recent network meta-analysis published in 2015 that indirectly compared the efficacy and safety of PDEIs, sildenafil had the greatest efficacy but the highest rate of overall adverse events, whereas tadalafil had intermediate efficacy and the lowest overall rate of all adverse events [15]. However, our study, which differs from previous systematic reviews, is the first meta-analysis to directly compare the efficacy and safety of sildenafil and tadalafil. PDE5-Is selectively block the PDE5 enzyme, which catalyses the hydrolysis of cGMP to GMP and thus prolongs erectile function [32]. PDEIs are the most commonly used drugs to treat ED, and the effects of the PDEIs used to treat ED have been investigated. The IIEF is a widely used, validated and self-administered questionnaire that has demonstrated a high degree of sensitivity and specificity for the assessment of ED [33]. First, we used the IIEF to evaluate the overall efficacy of sildenafil and tadalafil. Previous studies reported that PDEIs were more effective than placebos for treating ED and were generally safe and well tolerated [13]. Interestingly, our efficacy analysis revealed that sildenafil and tadalafil had equivalent abilities to improve IIEF-EF scores. No significant between-group differences for tadalafil and sildenafil were evident for the IIEF sexual desire, IIEF intercourse satisfaction and IIEF overall satisfaction domains. This finding is somewhat unexpected and may result from differences in the distribution of efficacy profiles within the different treatment strata assessed. However, a high level of significant between-trial heterogeneity was evident across the articles included in this meta-analysis, and the time or dose of administration of the PDEIs was diverse. The sensitivity analysis based on various exclusion criteria did not alter the forest plot and pooled effect, which strengthened our result.

ED involves both physiological and psychological factors, and increasing importance has recently been attached to the psychological aspects of ED diagnosis and management [34, 35]. PDEIs can improve a patient^’^s sexual confidence both directly, via improved EF, and indirectly, via improved spontaneity and diminished time concerns. Tadalafil significantly improves EF among psychogenic ED patients [36, 37]. Furthermore, pooled evidence suggests that tadalafil improves psychological outcomes, including the SEAR Confidence and Sexual Relationship scores and the total EDITS scores, to a greater degree than sildenafil. The SEAR is a subject-reported measure of psychosocial outcomes in men with ED, whereas the EDITS is a reliable and validated questionnaire used to assess patient satisfaction with ED treatment [38, 39]. The study by Eusebio Rubio-Aurioles revealed that the time concerns domain score was significantly lower for tadalafil once a day (OaD) compared with sildenafil PRN (on demand) treatment (*P* < 0.001) [23]. Thus, tadalafil improves sexual confidence more efficiently than sildenafil.

The adverse event rate for PDE5-Is is greater than the rate for placebos, but PDE5-Is are generally well tolerated for the treatment of ED. The most common adverse drug reactions include headache, flushing, nasal congestion, nasopharyngitis and dyspepsia [40], which are reflections of the vasodilatory effects on the capillary smooth muscle in other parts of the body. A trade-off network meta-analysis published by Yuan J suggested that PDE5-Is were generally safe and well tolerated and found no major difference in the safety profiles [13]. A later systematic review revealed that sildenafil had the highest rate of overall adverse events and that tadalafil had the lowest overall rate of all adverse events [15]. However, our meta-analysis suggests that the overall adverse event rate of tadalafil is equal to that of sildenafil, although the specific adverse events differ. For instance, there is no significant difference in the incidence of headache, dyspepsia, nasal congestion or nasopharyngitis between tadalafil and sildenafil, but tadalafil is associated with a higher incidence of myalgia and back pain and a lower occurrence of flushing than sildenafil. Furthermore, we found that the adherence and persistence rates of tadalafil were equal to those of sildenafil, which may be due to the medications’ similar efficacy, safety and tolerability properties.

In this study, we found that the majority of the patients and their partners preferred tadalafil over sildenafil. Currently, there are several effective and safe oral PDE5-Is for the treatment of ED, and various factors that are important to both the patient and his partner, such as biological, social, psychological and/or cultural factors, will influence the treatment choices [29]. Current ED guidelines also emphasize that the assessment of ED treatment must consider the effects on patient and partner satisfaction, which include psychosocial outcomes as well as efficacy and safety [1]. Therefore, this analysis evaluated the psychosocial outcomes and drug attributes of tadalafil versus sildenafil and found that tadalafil could improve psychosocial outcomes, including the SEAR Confidence and Sexual Relationship scores and the total EDITS scores, more than sildenafil. A previous study suggested that time concerns appeared to play a role in patient decisions regarding the ED treatment type, and the study by Eusebio Rubio-Aurioles reported that significant improvements in the PAIRS time concerns were observed after baseline. The improvement in the tadalafil group was superior to that of the sildenafil group, indicating that men who took tadalafil felt less time pressure and a reduced sense of urgency and had to do less planning before and during sexual encounters compared with those taking sildenafil [23]. Additionally, the mean half-life of sildenafil is approximately 4 h, with demonstrated improvement in EF for up to 8–12 h post-dose; conversely, the mean half-life of tadalafil is 17.5 h, with demonstrated improvement in EF for up to 36 h post-dose. The different pharmacokinetic profiles of tadalafil and sildenafil profiles grant patients more freedom to perform sexual intercourse with less need to plan ahead [41].

We must acknowledge the limitations of this meta-analysis. First, the studies included in the present meta-analysis were randomized or non-randomized controlled studies, and the blinding methods of these studies were not described in detail. We noted that the studies had flaws in quality, primarily in terms of the study design, patient selection, blinding and outcome data; thus, the results of this meta-analysis should be interpreted with caution. However, the results were strengthened by the fact that most of the included studies were randomized, multicentre trials. Second, high heterogeneity of IIEF-EF, IIEF intercourse satisfaction, men’s preference, adherence and persistence was found; this could be explained by the inclusion of studies with a low sample size and the various timing or doses of the PDEIs. A sensitivity analysis based on various exclusion criteria did not alter the forest plot and pooled effect, which strengthened our results. Third, when examining the risk of bias table across studies, we found publication bias for headache, flushing and nasal congestion, which limited the stated conclusions. Hence, a large-scale, high-quality, randomized, double-blind trial should be included in the meta-analysis to provide good evidence for the selection of specific PDEIs for the treatment of ED in the future. A comprehensive assessment of biases, study quality and heterogeneity should be a routine component of systematic reviews. Although the methods may be hampered by issues such as a low number of studies, large effect sizes and the design of the included studies, comprehensive assessments allow the reader to be more confident that the evidence presented is robust and that simple causes of confounding have been considered and explored. Without such an assessment, the conclusions of a systematic review may be weak and could lead to the application of inappropriate practices in healthcare settings.

## Conclusion

Tadalafil and sildenafil have been shown to exhibit comparable efficacy, safety and satisfaction for the treatment of ED, and tadalafil dramatically improved the psychological outcomes. The adherence and persistence rates for tadalafil and sildenafil were equal. Furthermore, both men and women preferred tadalafil to sildenafil for the treatment of ED. Thus, tadalafil may be a better choice for ED treatment. The findings of this meta-analysis will provide important evidence for the selection of PDEIs for the clinical treatment of ED.

## References

[1] Hatzimouratidis K, Amar E, Eardley I, Giuliano F, Hatzichristou D (2010). Guidelines on male sexual dysfunction: erectile dysfunction and premature ejaculation. Eur Urol.

[2] Oyelade BO, Jemilohun AC, Aderibigbe SA (2016). Prevalence of erectile dysfunction and possible risk factors among men of South-Western Nigeria: a population based study. Pan Afr Med J.

[3] Sathyanarayana RT, Darshan MS, Tandon A (2015). An epidemiological study of sexual disorders in south Indian rural population. Indian J Psychiatry.

[4] Ramezani MA, Ahmadi K, Ghaemmaghami A, Marzabadi EA, Pardakhti F (2015). Epidemiology of sexual dysfunction in Iran: a systematic review and meta-analysis. Int J Prev Med.

[5] Zhang Z, Li Z, Yu Q, Wu C, Lu Z (2015). The prevalence of and risk factors for prostatitis-like symptoms and its relation to erectile dysfunction in Chinese men. Andrology.

[6] Ayta IA, McKinlay JB, Krane RJ (1999). The likely worldwide increase in erectile dysfunction between 1995 and 2025 and some possible policy consequences. BJU Int.

[7] Ventimiglia E, Capogrosso P, Montorsi F, Salonia A (2016). The safety of phosphodiesterase type 5 inhibitors for erectile dysfunction. Expert Opin Drug Saf.

[8] Bruzziches R, Francomano D, Gareri P, Lenzi A, Aversa A (2013). An update on pharmacological treatment of erectile dysfunction with phosphodiesterase type 5 inhibitors. Expert Opin Pharmacother.

[9] Patel CK, Bennett N (2016). Advances in the treatment of erectile dysfunction: what’s new and upcoming?. F1000Res.

[10] Raheem AA, Kell P (2009). Patient preference and satisfaction in erectile dysfunction therapy: a comparison of the three phosphodiesterase-5 inhibitors sildenafil, vardenafil and tadalafil. Patient Prefer Adherence.

[11] Seftel AD (2002). Challenges in oral therapy for erectile dysfunction. J Androl.

[12] Evans JD, Hill SR (2015). A comparison of the available phosphodiesterase-5 inhibitors in the treatment of erectile dysfunction: a focus on avanafil. Patient Prefer Adherence.

[13] Yuan J, Zhang R, Yang Z, Lee J, Liu Y (2013). Comparative effectiveness and safety of oral phosphodiesterase type 5 inhibitors for erectile dysfunction: a systematic review and network meta-analysis. Eur Urol.

[14] Berner MM, Kriston L, Harms A (2006). Efficacy of PDE-5-inhibitors for erectile dysfunction. A comparative meta-analysis of fixed-dose regimen randomized controlled trials administering the International Index of Erectile Function in broad-spectrum populations. Int J Impot Res.

[15] Chen L, Staubli SE, Schneider MP, Kessels AG, Ivic S (2015). Phosphodiesterase 5 inhibitors for the treatment of erectile dysfunction: a trade-off network meta-analysis. Eur Urol.

[16] Jadad AR, Moore RA, Carroll D, Jenkinson C, Reynolds DJ (1996). Assessing the quality of reports of randomized clinical trials: is blinding necessary?. Control Clin Trials.

[17] Bai WJ, Li HJ, Jin JJ (2017). A randomized clinical trial investigating treatment choice in Chinese men receiving sildenafil citrate and tadalafil for treating erectile dysfunction. Asian J Androl.

[18] Buvat J, Buttner H, Hatzimouratidis K, Vendeira PA, Moncada I (2013). Adherence to initial PDE-5 inhibitor treatment: randomized open-label study comparing tadalafil once a day, tadalafil on demand, and sildenafil on demand in patients with erectile dysfunction. J Sex Med.

[19] Conaglen HM, Conaglen JV (2008). Investigating women’s preference for sildenafil or tadalafil use by their partners with erectile dysfunction: the partners’ preference study. J Sex Med.

[20] Eardley I, Mirone V, Montorsi F, Ralph D, Kell P (2005). An open-label, multicentre, randomized, crossover study comparing sildenafil citrate and tadalafil for treating erectile dysfunction in men naive to phosphodiesterase 5 inhibitor therapy. BJU Int.

[21] Govier F, Potempa AJ, Kaufman J, Denne J, Kovalenko P (2003). A multicenter, randomized, double-blind, crossover study of patient preference for tadalafil 20 mg or sildenafil citrate 50 mg during initiation of treatment for erectile dysfunction. Clin Ther.

[22] Hatzimouratidis K, Buvat J, Buttner H, Vendeira PA, Moncada I (2014). Psychosocial outcomes after initial treatment of erectile dysfunction with tadalafil once daily, tadalafil on demand or sildenafil citrate on demand: results from a randomized, open-label study. Int J Impot Res.

[23] Rubio-Aurioles E, Porst H, Kim ED, Montorsi F, Hackett G (2012). A randomized open-label trial with a crossover comparison of sexual self-confidence and other treatment outcomes following tadalafil once a day vs. tadalafil or sildenafil on-demand in men with erectile dysfunction. J Sex Med.

[24] Tolra JR, Campana JM, Ciutat LF, Miranda EF (2006). Prospective, randomized, open-label, fixed-dose, crossover study to establish preference of patients with erectile dysfunction after taking the three PDE-5 inhibitors. J Sex Med.

[25] von Keitz A, Rajfer J, Segal S, Murphy A, Denne J (2004). A multicenter, randomized, double-blind, crossover study to evaluate patient preference between tadalafil and sildenafil. Eur Urol.

[26] Li HJ, Bai WJ, Dai YT, Xu WP, Wang CN (2016). An analysis of treatment preferences and sexual quality of life outcomes in female partners of Chinese men with erectile dysfunction. Asian J Androl.

[27] Cairoli C, Reyes LA, Henneges C, Sorsaburu S (2014). PDE5 inhibitor treatment persistence and adherence in Brazilian men: post hoc analyses from a 6-month, prospective, observational study. Int Braz J Urol.

[28] Stroberg P, Murphy A, Costigan T (2003). Switching patients with erectile dysfunction from sildenafil citrate to tadalafil: results of a European multicenter, open-label study of patient preference. Clin Ther.

[29] Ahn TY, Lee SW, Kim SW, Yang DY, Park NC (2007). Treatment preferences in men with erectile dysfunction: an open label study in Korean men switching from sildenafil citrate to tadalafil. Asian J Androl.

[30] El-Meliegy A, Rabah D, Al-Mitwalli K, Mostafa T, Hussein T (2013). A 6-month, prospective, observational study of PDE5 inhibitor treatment persistence and adherence in Middle Eastern and North African men with erectile dysfunction. Curr Med Res Opin.

[31] Taylor J, Baldo OB, Storey A, Cartledge J, Eardley I (2009). Differences in side-effect duration and related bother levels between phosphodiesterase type 5 inhibitors. BJU Int.

[32] Rizio N, Tran C, Sorenson M (2012). Efficacy and satisfaction rates of oral PDE5is in the treatment of erectile dysfunction secondary to spinal cord injury: a review of literature. J Spinal Cord Med.

[33] Tang Z, Li D, Zhang X, Yi L, Zhu X (2014). Comparison of the simplified International Index of Erectile Function (IIEF-5) in patients of erectile dysfunction with different pathophysiologies. BMC Urol.

[34] Rew KT, Heidelbaugh JJ (2016). Erectile dysfunction. Am Fam Physician.

[35] Wang W (2011). Psychological factors involved in erectile dysfunction. Zhonghua Nan Ke Xue.

[36] Sontag A, Ni X, Althof SE, Rosen RC (2014). Relationship between erectile function and sexual self-confidence: a path analytic model in men being treated with tadalafil. Int J Impot Res.

[37] Li G, Lan H, Liang J, Zhang C, Huang C (2017). Efficacy of tadalafil de-escalation in the treatment of psychogenic erectile dysfunction. Urol Int.

[38] Albertsen PC (2005). Development and validation of the self-esteem and relationship (SEAR) Questionnaire in erectile dysfunction. J Urol.

[39] Althof SE, Corty EW, Levine SB, Levine F, Burnett AL (1999). EDITS: development of questionnaires for evaluating satisfaction with treatments for erectile dysfunction. Urology.

[40] Huang SA, Lie JD (2013). Phosphodiesterase-5 (PDE5) inhibitors in the management of erectile dysfunction. P T.

[41] Bai WJ, Li HJ, Dai YT, He XY, Huang YR (2015). An open-label, multicenter, randomized, crossover study comparing sildenafil citrate and tadalafil for treating erectile dysfunction in Chinese men naive to phosphodiesterase 5 inhibitor therapy. Asian J Androl.

